# Validation of Physical Activity Correlates Questionnaire from Social Ecological Model in College Students

**DOI:** 10.3390/jcm12030777

**Published:** 2023-01-18

**Authors:** Meirong Huang, Haichun Sun, Han Chen, Yanping Zhang, Kaitlyn Adams, Zan Gao

**Affiliations:** 1 School of Physical Education, China University of Mining and Technology, Xuzhou 221116, China; 2 College of Education, University of South Florida, Tampa, FL 33620, USA; 3 Department of Teacher Education-Kinesiology and Physical Education, Valdosta State University, Valdosta, GA 32698, USA; 4 School of Kinesiology, University of Minnesota-Twin Cities, Minneapolis, MN 55455, USA

**Keywords:** facilities, interest and values, media, physical activity determinants, social support

## Abstract

More than 50% of Chinese college students rarely engage in any type of physical activity (PA). The literature shows several issues in college students’ health due to lack of PA. Promoting college students’ PA participation across the country has become a challenging task. This study aims to create a questionnaire into the correlates that affect Chinese college students’ engagement in leisure time PA. We first determined the factor structure of college students’ PA engagements. Then, we tested and verified the reliability and validity of the factor structure of the questionnaire. The Chinese college students’ PA correlates questionnaire was designed according to the Social Ecological Model. Exploratory factor analysis (EFA) extracted all the included items, whereas confirmatory factor analysis (CFA) verified the validity of the model. We recruited 1290 college students to complete the questionnaire. A second-order structural model was constructed. Specifically, the first-order included individual, social, and environmental as dimensions, while the second-order had interest, media, social support, traditional culture, facilities, and environment as factors. The six factors have polymerized 22 items. The model demonstrated a good fit (X^2^/*df* = 1.4; CFI = 0.98; GFI = 0.94; RMSEA = 0.02; SRMR = 0.05). In conclusion, the newly established questionnaire of college students’ PA correlates is reasonable, and the results of factors reliabilities and model fit are acceptable.

## 1. Introduction

The health benefits of regular participation in moderate to vigorous physical activity (MVPA) have been well-documented among various populations. A study of the sedentary and physical activity (PA) behaviors of adults suggests that participation in MVPA has an independent association with a lower risk of chronic diseases such as type 2 diabetes, cardiovascular disease, and obesity [1]. However, despite the widely acknowledged health benefits of MVPA, research has shown that college students worldwide lack PA involvement. For example, the American College of Health Association-National College Health Assessment III Reference Group Data Report-Fall 2021 revealed that 33.4% of U.S. college students had participated in 0 min of vigorous PA during the past seven days. In addition, 42.5% of college students reported not participating in any kind of exercises to strengthen or tone their muscles [2].

Similar PA patterns were also identified among Chinese college students. Research evidence has shown that more than 60% of Chinese college students did not participate in regular PA and some (21.85%) even lived an almost completely sedentary lifestyle [3,4,5,6,7,8,9]. A recent study reported that 64.8% of Chinese freshmen college students are sedentary [10]. Many Chinese college students did not meet the minimum PA levels recommended by the World Health Organization (WHO), which recommends 150 to 300 min of moderately intense exercise or 75 to 150 min of high-intensity aerobic exercise every week [11,12]. Considering the health risks associated with insufficient PA, it is imperative to identify effective strategies to promote a physically active lifestyle among Chinese college students. Therefore, the initial step is to explore the factors that impact Chinese college students’ PA participation.

Researchers have provided important implications concerning children and adolescents’ PA and PA correlates in China [13,14,15,16]. However, previous studies assessing PA correlates have some limitations concerning the theoretical framework and/or methodological issues. For example, some studies were conducted with a small sample size, and without a confirmatory factor analysis (CFA) and/or invariances test. In addition, most research examining the PA correlates influencing Chinese college students’ PA are mainly based on individual or social level theoretical frameworks, and factors from an organization dimension were neglected [17,18]. China is a collectivist country, which means that groups in society may play a vital role in influencing people’s PA behavior [19]. Given the fact that the number of Chinese college students reached 44.3 million in 2022 [20], the world’s largest college student population, it is important to understand the factors associated with Chinese college students’ PA behaviors in order to promote a physically active lifestyle and to help health professionals and practitioners design appropriate interventions in the future. 

**Figure 1:**
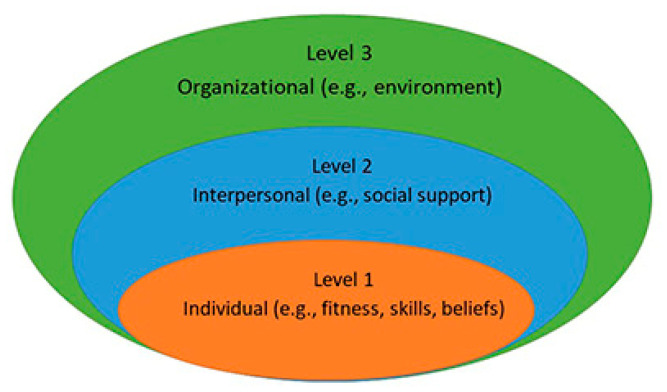
Social ecological model.

Among the theories on individuals’ health-related behaviors, the Social Ecological Model (SEM) has been considered as one of the most salient and effective frameworks to explain how intrapersonal factors (e.g., psychology and physiology) interact with social (e.g., social support, media, and policy) and environmental factors (e.g., community and school) [21,22,23]. SEM provides a framework (see Figure 1) for researchers to understand the complex interactions among multiple factors and to answer specific questions in particular circumstances, as well as to identify health issues in certain domains (e.g., PA) of specific populations to design more tailored interventions. It has been evident that the multiple levels of factors (individual, interpersonal, and organizational) have direct and indirect influences on individuals’ PA among different populations [24,25,26,27]. For example, Zhang et al. (2018) examined the effects of individual, social, and physical environmental factors on college women’s (N = 235) PA and health-related quality of life; they found that self-efficacy and crime safety were significantly related to PA involvement [28]. Therefore, the researchers suggested that PA or health professionals need to foster a safe environment, enhance self-efficacy, and design enjoyable activities to engage college female students’ PA participation. Research has also suggested that SEM-based PA interventions could be effective. Simon et al. (2014) investigated whether the efficacy of a school-based socio-ecological PA program persisted 30 months after intervention cessation. They found a significant reduction effect of the intervention on children’s body mass index (BMI) measured 30 months after the completion of the original 4-year trial, especially in the sedentary and least wealthy subgroups [29]. Thus, we will adopt SEM as the theoretical framework to develop the PA correlates questionnaire in this study.

The primary purpose of this study, therefore, was to develop and validate a questionnaire to examine the correlates affecting Chinese college students’ PA participation. The study also aimed to increase our understanding of the correlates affecting Chinese college students’ PA behaviors from the Social Ecological perspective. We hypothesize that the newly established Physical Activity Correlates Questionnaire―Chinese version would be valid and reliable. The findings of this study may have the potential to make significant contributions to the literature and practice. Specifically, our study could provide a valid and reliable instrument that identifies Chinese college students’ PA correlates.

## 2. Methods

The validation process included three steps. Firstly, we created items based on the literature and specified the three dimensions according to the SEM. Secondly, we sent the questionnaire to five experts in the fields of psychology, management, and physical education to examine the content validity. In this step, the experts also reviewed the items and shared their corresponding dimensions. Thirdly, we randomly split the total sample into two sub-samples with each one used to conduct either EFA or CFA test to test the construct validity of the questionnaire.

### 2.1. Participants

We recruited 1290 college students from ten universities in Eastern China to complete the questionnaire. The sample included 426 female students (33%) and 864 male students (67%). The average age was 19.70 ± 0.052 years old. All participants were full-time college students and Han ethnicity.

### 2.2. Questionnaire Development

The initial items in the questionnaire were written guided by the three dimensions of the SEM model: individual, social, and environmental. We went through several rounds of in-depth deliberation and revision to ensure consistency between the items and their respective dimensions. We also conducted focus group interviews (*n* = 38) to clarify any confusion about the language used in the questionnaire. Necessary revisions were made to items due to language issues, according to the information collected from focus group interviews.

### 2.3. Content Validation

The purpose of this step was to consider whether the items we developed were able to accurately reflect the three dimensions of the SEM. The expert judgmental method was thus used to examine the content validity of the questionnaire [30]. We identified five experts who had expertise in the SEM as related to PA behaviors. We sent the draft to the experts soliciting their evaluations of the items by a social networking site, QQ (Tencent Company, Shenzhen, China). We revised the questionnaire according to the experts’ feedback and recommendations and sent the revisions back to them for further review. Two rounds of review-revision were made to address the experts’ comments and concerns.

### 2.4. Construct Validation

Construct validation is a theory-driven process. The purpose of construct validation is to put the measure of a construct in a nomological network and test how the construct relates to other variables according to a theoretical framework or frameworks [31]. In other words, construct validation of an instrument is to examine “the extent to which its observed associations with measures of other variables match theoretical predictions about how it should be associated with those variables” [32]. To test the construct validity, we recruited a sample of college students and collected their responses to the College Students’ PA Correlates Questionnaire that had been content validated.

### 2.5. Data Collection

Before the data collection, we obtained approval from the university’s research office (equivalent to Institutional Review Board) and received students’ consent forms. The questionnaires were distributed to participants through the two most popular social networking platforms in China, WeChat and QQ, from 4 September 4 to 11 October 2019. We created an account using Questionnaire Star and designed the questionnaire in accordance with the procedure and formed a network link. The network link was then forwarded to the participants through WeChat and QQ. Data collected through these sites were automatically transferred to SPSS.

### 2.6. Data Analysis

Among the 1290 responses, 39 were deemed invalid and therefore deleted from the final data set. An analysis suggested that the invalid data were random and further analyses were not warranted. The final sample of 1251 (66.7% male) participants were randomly divided into two independent subsamples in SPSS. The first group was used for the EFA test (*n* = 610, 66.9% male) and the second group was used for the CFA test (*n* = 641, 66.6% male).

The construct validation included two steps: dimension identification and dimension confirmation. Specifically, the convergent and discriminant validity and reliability were examined by EFA [33]. The SPSS version 22.0 was used to conduct an EFA test in which the principal axis factoring extraction method with Promax (oblique) rotation on all indicators was applied. Both scree plot and Kaiser’s criteria were utilized to determine the number of factors extracted with an eigenvalue greater than 1 [34,35]. In addition, items with a loading higher than 0.40 were retained in the model.

In the dimension confirmation process, CFA was carried out to examine the factor structure of the college students’ PA correlates questionnaire. The CFA test was conducted with AMOS version 22.0 on the second group using the following steps. In the first step, we developed the multifactor solution model with all factors correlated with one another. The multivariate normality test on the data was analyzed in which the cutoff point of 0.50 was utilized to determine the violation of multivariate normality distribution. In case of the violation, the Bollen-Stine bootstrapping method (*n* = 2000 resample) was used to adjust the values of the inflated Chi-square as well as various fit indices [36]. Model fit was determined by various fit indices including Chi-square (X^2^)/degree of freedom (*df*), comparative fit index (CFI), goodness-of-fit index (GFI), root-mean-square error of approximation (RMSEA), and standardized root-mean-square residual (SRMR). Standardized factor loading was set to be larger than 0.50 [37]. In the second step, a discriminant validity test of the model was conducted to examine the degree to which each factor was distinctive from the other factors. According to Bagozzi, Fornell and Larker (1981), the value of the square root of the average variance extracted (AVE) of each construct in the model should be higher than its correlations among other constructs, which indicates the establishment of the discriminant validity for all factors [38]. In the third step, measurement invariance tests across genders were conducted with the total sample using progressive restrictive stages at both configural (equal factor structure), full metric (equal factor loadings), and full scalar (equal factor loadings and items intercepts) levels [39]. The establishment of the configural model was determined by acceptable model fit. The establishments of both metric and scalar invariances were assessed by checking the value of CFI change in which CFI change < 0.01 indicates the invariance at either full metric or full scalar level [40].

## 3. Results

### 3.1. Questionnaire Development and Content Validation

According to the SEM, we originally created a questionnaire with a total of 45 items. After deliberation and group discussion, we retained 41 items. All the items were randomly placed on the questionnaire and then sent to the expert panel for the content validity check. Five experts provided independent but consensual feedback suggesting that six items should be removed and only retained as the demographic questions. The experts also considered three items irrelevant to the SEM and suggested that those items should be deleted. Thus, 32 items were retained after two rounds of review.

### 3.2. Construct Validation

*EFA results.* Table 1 shows all six factors, as well as the items’ standardized factor loadings after rotation. Outcomes from both the Kaiser-Meyer-Olkin test (KMO = 0.91) and the Barlett’s test of sphericity (X^2^ (496) = 7204.1, *p* < 0.001) supported the use of the EFA test in the first group. Findings from the EFA test generated six factors that had eigenvalues larger than 1 (Kaiser’s recommendation). All six factors in combination explained a total of 54.6% of the variance. Results from the scree plot also suggested retaining these six factors. The six factors were consistent with the theoretical assumption of the model and represented the three dimensions of individual, social, and environmental. Specifically, these factors are PA interest and values, media, social support, Chinese traditional culture, facilities, and weather.

*Reliability and validity.* Given the nature of the latent variable modeling context, we calculated the composite reliability of the measurement properties of the scale to provide a rigorous assessment of the internal reliability of the measure [41,42]. The results showed these factors have adequate composite reliabilities: PA interest and values is 0.85, media is 0.73, social support is 0.80, weather is 0.63, Chinese traditional culture is 0.61, and facilities is 0.69 [43].

*CFA results.* CFA was conducted to test the tenability of the construct structure revealed in EFA as well as measurement invariance across. The CFA model was developed based on the EFA test for the second group. The initial normality test revealed that data violated the multivariate normality distribution. Accordingly, the Bollen-Stine bootstrapping method (*n* = 2000) was used for adjustment of the inflated Chi-square. After adjustment, the six-model demonstrated good model fit (X^2^/*df* = 1.4; CFI = 0.98; GFI = 0.94; RMSEA = 0.02; SRMR = 0.05). The CFA test also found that most of the items’ standardized factor loadings were larger than 0.50 (except for one item in which the loading was very close to 0.50; see Table 2). Findings from the discriminant validity test revealed that among all six factors, five of them could be distinct from the other factors and met the discriminant validity. However, there was one factor (Media) that may have a potential to overlap with two other factors (Social support, Interest) even though the square root of AVE for Media is just slightly larger than its correlations with Social support and Interest (see Table 3).

A measurement invariance test across gender was conducted using the total sample. Findings showed that the invariance test was established at configural level as evidenced by acceptable model fit (X^2^/*df* = 3.07; CFI > 0.90; RMSEA < 0.06). In addition, measurement invariance across gender was established at a full metric level. However, full scalar invariance between gender was not established since the value of CFI change was larger than 0.01 (see Table 4). 

## 4. Discussion

The study developed a questionnaire based on the SEM with two orders, three dimensions, and six factors. By comparison, Zhang and colleagues’ questionnaire with 47 items, which explored adolescents’ interest and values of PA, health-related fitness knowledge, PA participation, school and family support, environmental factors, and PA barriers [44], is more general. The present study, on the contrary, only focuses on the population of Chinese college students and their PA correlates. The questionnaire is simpler and clearer in capturing what impacts college students’ PA. Specifically, the 22 final items with six factors of PA, namely PA interest and values, media, social support, weather, traditional Chinese culture, and facilities, are more efficient and applicable to the target population in this study.

Recently, Si, Wang, Kim, and Zhu (2017) established a questionnaire including four subscales and 13 factors to explore adolescents’ in-school PA determinants [45]. The 13 factors have polymerized 62 final items. Among them, the friend support subscale contained two factors; the teacher support subscale included three factors; and the environment and policy subscale consisted of three factors, respectively. On the other hand, our questionnaire has good surveying features, is concise, less time-consuming, and the delivery is simple. This questionnaire can be used by researchers, health professionals, and practitioners to investigate factors affecting college students’ PA behaviors. Building upon our findings, theoretical guidance and practical guidance could be provided to college students to promote PA and school sports. 

The study findings have practical implications to the relevant end user. For example, our data indicated that the policy factor could not be clustered to any one dimension that affects college students’ PA behaviors. It has been reported that the Chinese government issued a series of policies to promote college students’ PA behaviors, but that the effect was insignificant [46]. The relevant end user should pay attention to who the policies target when designing PA interventions for college students. The government and schools should create mutually beneficial environments to encourage students and help motivate them to improve their levels of PA participation. If the universities construct and/or renovate their sports facilities, they should emphasize the convenience of sports facilities concerning location. The proximity to places to be active and the availability of places for activity and recreation are positively related to PA behavior. Adults who live in more walkable communities are more physically active than those who live in less walkable communities [47].

The establishment of the configured invariance across gender implies that both males and females interpret the College Students’ Physical Activity Correlates Questionnaire with the same patterns. Specifically, the same factors and items were identified by both genders. The establishment of the full metric invariance of this questionnaire across gender suggests that the association between the questionnaire and other variables can be compared between genders.

The study followed the questionnaire revision procedure strictly and adopted a large sample size to conduct analysis using two sub-samples via EFA and CFA. However, several limitations of the present study should be identified. Firstly, the data of this study were collected primarily via remote modality before the COVID-19 pandemic. Secondly, the data were only collected from ten universities in Eastern China while there are 2956 universities overall and eight regions in China. However, the students of the ten universities in the Eastern China region come from many provinces in China, which may mitigate this limitation to a certain degree. Thirdly, the questionnaire developed in this research was based on a Chinese cultural background and written in Chinese. Future studies may need to perform CFA before an English version is used for testing. Additionally, college students’ PA behaviors might be affected by other factors, such as ethnicity [48]. This study did not explore the differences among the 56 ethnicities in China and only targeted Han ethnicity. Future studies may recruit more diverse college student populations from most, if not all, ethnicities in the nation.

Overall, the College Students’ PA Correlates Questionnaire provides a convenient and effective tool for the measurement of college students’ PA correlates and determinants in China, which provides substantial support for our research hypothesis. Scholars and educators may find the core factors that promote college students’ PA participation. The findings of the present study have a number of potential implications for health professionals and practitioners working with college students’ PA promotion. Given that regular participation in PA has numerous health benefits and is highly recommended by the World Health Organization [49,50,51,52,53,54], further studies are needed to investigate the feasibility of effective and innovative interventions to promote college students’ PA participation [55].

## 5. Conclusions

A multi-phase instrument development procedure was used for this study. The questionnaire consists of 22 items on PA correlates for Chinese college students. An expert review panel validated the content validity, and a two-step cross-sectional testing procedure with a split-sample method was conducted to validate the construct validity. EFA and CFA confirmed the three-dimension six-factor construct structure: PA interest and values, media, social support, weather, traditional Chinese culture, and facilities. Based upon the content validation, construct validation, and reliability tests, we found the newly established questionnaire to be reasonable, and the results of factor reliabilities and model fit are acceptable. The findings suggest that the newly established questionnaire of college students’ PA correlates is reasonable, and the results of factors reliabilities and model fit are acceptable. Therefore, we recommend the use of this questionnaire to assess Chinese college students’ PA correlates in future studies.

## Figures and Tables

**Table 1:** Outcomes from EFA test based on the first group (*n* = 610).

	Factors					
	1	2	3	4	5	6
*Interest*						
1 I think physical exercise is fun (int1)	0.70					
24 I feel very happy doing physical exercises (int2)	0.76					
31 I think physical exercise can make me happy (int3)	0.69					
5 I think physical exercise can promote my interaction with other people (int4)	0.52					
10 I feel physical exercise makes me very energetic (int5)	0.68					
*Social Support*						
3 My classmates and friends often do physical exercises (soc1)			0.75			
11 When I am with my classmates and friends, my classmates or friends often do physical exercises with me (soc2)			0.69			
13 My classmates and friends often discuss topics related to physical exercise activities with me (soc3)			0.68			
22 My classmates and friends often encourage me to keep exercising (soc4)			0.60			
*Media*						
2 Publicity on physical exercise in social media affects my physical exercise (med1)		0.57				
25 Publicity about physical exercise on school website, radio station, campus slogan, etc. promotes me to exercise (med2)		0.70				
32 The promotion of physical exercise on social network platforms such as WeChat, Weibo, and QQ promotes me to exercise (med3)		0.59				
*Facilities*						
9 It doesn’t take long to reach my school’s sports facilities (fac1)				0.45		
15 The school sports venues and facilities can meet my needs for physical exercise (fac2)				0.56		
26 Sports facilities of our school include indoor stadiums, outdoor stadiums, swimming pools/halls, tennis courts, etc. (fac3)				0.57		
30 I have convenient transportation to school sports facilities (fac4)				0.77		
*Traditional culture*						
16 In order to study, I hardly have time for physical exercise (tra2)					0.52	
19 I think people who exercise regularly have relatively well-developed limbs and simple minds (tra3)					0.73	
21 I think learning is more important than physical exercise (tra4)					0.45	
*Environment*						
6 I don’t do physical exercises if it’s too cold or too hot (env1)						0.46
20 I don’t do physical exercises in severe weather such as wind, rain and snow (env4)						0.69
27 I don’t do physical exercises if the air quality is not good (env6)						0.62

Note. Cross loadings < 0.40 were not shown.

**Table 2:** Standardized factor loadings from CFA test based on the second group (*n* = 641).

Items		Factors	Standardized Factor Loadings
1 I think physical exercise is fun (int1)	<---	Interest	0.70
24 I feel very happy doing physical exercises (int2)	<---	Interest	0.76
31 I think physical exercise can make me happy (int3)	<---	Interest	0.80
5 I think physical exercise can promote my interaction with other people (int4)	<---	Interest	0.64
10 I feel physical exercise makes me very energetic (int5)	<---	Interest	0.71
3 My classmates and friends often do physical exercises (soc1)	<---	Social support	0.71
11 When I am with my classmates and friends, my classmates or friends often do physical exercises with me (soc2)	<---	Social support	0.78
13 My classmates and friends often discuss topics related to physical exercise activities with me (soc3)	<---	Social support	0.79
22 My classmates and friends often encourage me to keep exercising (soc4)	<---	Social support	0.62
2 Publicity on physical exercise in social media affects my physical exercise (med1)	<---	Media	0.65
25 Publicity about physical exercise on school website, radio station, campus slogan, etc. promotes me to exercise (med2)	<---	Media	0.68
32 The promotion of physical exercise on social network platforms such as WeChat, Weibo, and QQ promotes me to exercise (med3)	<---	Media	0.70
9 It doesn’t take long to reach my school’s sports facilities (fac1)	<---	Facilities	0.49
15 The school sports venues and facilities can meet my needs for physical exercise (fac2)	<---	Facilities	0.67
26 Sports facilities of our school include indoor stadiums, outdoor stadiums, swimming pools/halls, tennis courts, etc. (fac3)	<---	Facilities	0.54
30 I have convenient transportation to school sports facilities (fac4)	<---	Facilities	0.66
16 In order to study, I hardly have time for physical exercise (tra2)	<---	Traditional culture	0.67
19 I think people who exercise regularly have relatively well-developed limbs and simple minds (tra3)	<---	Traditional culture	0.52
21 I think learning is more important than physical exercise (tra4)	<---	Traditional culture	0.52
6 I don’t do physical exercises if it’s too cold or too hot (env1)	<---	Environment	0.60
20 I don’t do physical exercises in severe weather such as wind, rain and snow (env4)	<---	Environment	0.75
27 I don’t do physical exercises if the air quality is not good (env6)	<---	Environment	0.55

**Table 3:** Results from the discriminant validity test based on the second group (*n* = 641).

	Environment	Traditional Culture	Facilities	Media	Social Support	Interest
Environment	**0.63**					
Traditional culture	0.34	**0.57**				
Facilities	0.09	−0.35	**0.59**			
Media	−0.13	−0.16	0.47	**0.68**		
Social support	−0.30	−0.20	0.44	0.72	**0.73**	
Interest	−0.17	−0.36	0.56	0.71	0.67	**0.72**

Note. Square roots of AVE are bold on diagonals; off diagonals are correlations among latent factors.

**Table 4:** Results from measurement invariances tests across gender based on the full sample (N = 1251; *n* for males = 835; *n* for females = 416).

	X^2^/*df*	CFI	RMSEA	ΔCFI
Invariance across gender				
Configural level	3.07	0.905	0.041	
Full metric level	3.02	0.903	0.040	0.002
Full scalar level	3.49	0.874	0.045	0.029

## Data Availability

The data presented in this study are available on request from the corresponding author. The data are not publicly available due to privacy.
